# Extra-Uterine Pregnancy

**Published:** 1903-08-22

**Authors:** 


### Hospital Clinics and Medical Progress.

### EXTRA-UTERINE PREGNANCY.

The number of cases of extra-uterine pregnancy encountered in the course of ordinary practice is small, and the majority of practitioners probably could not recall more than three or four occasions on which they have consciously had to deal with this condition. It is believed that impregnation, as a rule, takes place in close contiguity to the ovary, whence the fertilised ovum travels down the fallopian tube to the site of its natural development, the uterine cavity. It would appear that its progress from the ovary to the uterus normally occupies a period of from eight to ten days, and that during it the ovum undergoes some development so that it reaches the uterus as a gelatinous mass about the size of a pea.

### Its Frequency.

During the whole of these eight or ten days the process is liable to arrest, and it is unlikely that the number of cases in which arrest followed by development or ectopic gestation is recognised, even by the most careful observers under the most favourable circumstances, bears any true proportion to the number of cases in which it really occurs. It is quite natural that this should be the case since it is probable that in the majority of arrested ova vitality ceases long before any of the more marked symptoms of pregnancy have had time to develop. Under such circumstances the ovum is probably either absorbed *in situ* or discharged in the course of what is regarded by the patient as the occurrence of a retarded or unusually disturbing period; while it would even appear that rupture of a tube and cessation of the gestation may occur without giving rise to truly pathognomonic indications, the slight shock, etc., being ascribed to other causes. It is certainly only in those cases in which the ovum has survived the risks of its abnormal situation sufficiently long for ordinary symptoms of pregnancy to occur and for the ovum itself to attain considerable development that the condition is capable of diagnosis even by the most careful observer. Judging, moreover, from the number of cases in which fœtal structures have been found in the course of operations for conditions which had not been ascribed in any way to the existence of a previous pregnancy, it would appear that extra-uterine pregnancy, sometimes at least, lasts for a very considerable time, and terminates in death of the fœtus, without the patient being aware of her condition and without any symptoms occurring on the death of the fœtus of a sufficiently characteristic nature to draw attention to what has occurred.

### Theories as to its Cause.

The proportion, therefore, of normal to extra-uterine pregnancies must be considered a matter of uncertain speculation and probably will always remain so, and the same remark applies to some extent to a consideration of the causes to which arrest of the ovum is due and of what is its habitual or commonest result. Almost all writers on obstetrics discuss the subject at some length, but it has received special attention at the hands of Lusk,[1] Lawson,[2] Tait, Webster,[3] Schmool,[4] Kelly,[5] and others. The theories formerly held upon the subject were chiefly of a mechanical nature. It was considered that failure of the ovum to reach the uterus was sufficiently explained by abnormal conditions within or without the tube of such nature as either to narrow its lumen or, according to Lawson Tait, destroy the ciliated epithelium which normally directs the ovum outwards towards the uterus. It is conceivable that the lumen might be sufficiently patent to allow of the occasional passage upwards of spermatozoa and yet be too small to permit the passage downwards of the larger and growing ovum. Ordinary salpingitis would be likely to bring about the loss of cilia to which Tait attached such importance, and might also cause thickening of the lining membrane. Twisting or bending of the tube through external adhesions would produce the same effect. More recently these theories, though supported by the fact that ectopic gestation is commonest in women who are either sterile or who have not borne children for many years, have been deemed insufficient. It is recognised more completely that genetic changes take place in the uterine mucosa exceedingly early after fertilisation, and that these decidual changes are primarily the cause rather than the consequence of the adhesion of the fertilised ovum; which in their absence would presumably not be arrested at all but pass out of the uterus and out of the body altogether. Normally these decidual changes are limited to the uterine cavity, but it has been shown that occasionally, at least, they occur not only in this situation but in the tubes and elsewhere. The tendency, therefore, of more recent observers is to attach more weight to these occasional extensions of the genetic changes to the tubes than to any mechanical theory of causation. The most, however, than can be said with certainty is that if such abnormal extension is not necessary to arrest of the ovum it is in any case essential for the prolongation of its vitality for more than a few days.

### Favourable and Unfavourable Conditions.

To these more or less speculative considerations Ward Cousins[6] has lately added one on the conditions which are favourable to the prolongation of an ectopic gestation beyond those two or three months which commonly see its close even in recognised cases, apart from those probably much more common cases in which the gestation aborts unrecognised at a very much earlier period. In Ward Cousins' opinion one of the conditions most favourable to the vitality of an ectopic fœtus is the continued mobility of the gestation sac.

During early stages the growing ovum and its sac are always very mobile and occupy different situations, in front, to one side, behind the uterus, or deep in Douglas' pouch. So long as mobility continues all may go well, but if it be interfered with, for instance, by impaction of the growing sac within the pelvis or through its adhesion to surrounding organs, the chances of continued vitality are materially diminished.

Another favourable condition is gradual and continuous expansion of the tube which forms the original sac. In cases which advance favourably this appears to undergo a slow process of stretching without any laceration of the original fibrous structure. Plastic exudation occurs around it, and even if the original structure does in any part give way, such rupture takes place into what is a closed and limited cavity, so that there is no important hæmorrhage, and the placental circulation is not interrupted.

A third favourable condition is early ascent of the gestation sac in the abdominal cavity. It may advance upwards almost vertically or assume an oblique position, carrying before it first the peritoneal covering of the pelvic viscera and then that of the anterior abdominal wall. In other cases, though the ascent may be successfully commenced, it may remain incomplete, the sac stretching backwards towards the spine, the broad ligament condensing and thickening around it. In such case, as well as in those in which the sac has dropped into the deeper parts of the pelvis and failed to rise out of it at all, comparatively early destruction of the fœtus must inevitably occur owing either to its compression by surrounding structures or to interference with the placental circulation.

### Position of the Placenta.

Probably, however, the duration of the pregnancy is more influenced by the position of the placenta than by any other factor. When the ovum becomes arrested in the tube and a vital connection is established, placental formation rapidly progresses, and may occur either below, above, or to the side of the growing ovum. In the former position it is capable of very little displacement, and practically cannot contribute at all towards the building up of the wall of the gestation sac. The latter, therefore, as it rises out of the pelvis has no further support than what it gains from a layer of peritoneum. In such case rupture is naturally likely to occur early, but nevertheless cases are on record in which, in spite of the extreme thinness of the sac, gestation has proceeded practically to full term. In the second position, that in which the placenta is uppermost, the latter expands and forms the upper part of the sac, serving to give it strength. Early rupture is therefore somewhat less liable to occur, but, on the other hand, the placental tissue is subjected to the constant pressure of the growing fœtus on one side and on the other to the varying tension of the surrounding viscera, and this continuously increasing but ever-varying pressure eventually leads to structural changes and consequent fœtal death. The most favourable condition of all is that in which the placenta occupies a lateral position in relation to the sac. In this it is capable of extension and prolongation upwards for many inches in the abdomen. It thus not only helps to strengthen the wall of the gestation sac, but at the same time remains in such close proximity to the large pelvic vessels that free circulation is ever secure.

### General Considerations.

The above are the conditions which, in Ward Cousins' view, mainly influence the progress of an ectopic gestation, but how any of them are capable of precise clinical recognition is doubtful. If precisely ascertained they would doubtless form a useful guide to the question of delaying or hurrying on any operative measures that might be foreshadowed. To this special consideration of the conditions favourable to prolonged gestation Ward Cousins adds certain general observations. Even in watched cases of pregnancy, correct and early diagnosis of an abnormal site is often impossible, while in the majority of cases of abdominal pregnancy, sudden rupture of the tube is often the first noted indication of anything being wrong. The diagnosis then rests upon a recognition of intra-peritoneal rupture and hæmorrhage, but since abdominal enlargement is often inappreciable and palpation is insufficient to establish the diagnosis of hæmorrhage, chief reliance must be placed upon the symptoms of sudden collapse associated with acute abdominal pain and tenderness. The urgency of the symptoms always corresponds with the suddenness and extent of the rupture. In a large proportion of all cases the urgency is not extreme and laparotomy not essential, for in early cases the embryonic structures become enveloped in effused blood and fixed by protective exudation, and in a few months absorbed. This favourable result is, he considers, possible up to the middle or end of the third month, but not later, since by that time ossification has commenced and the placenta is fully formed. In any case where the symptoms of pregnancy are associated with the occurrence of fitful and irregular hæmorrhage, obscure pelvic pains, the presence of a swelling adjacent to, or continuous with, the uterus, and well-marked changes in the breasts, he considers there is sufficient *primâ facie* evidence of an unruptured tubal pregnancy to justify an abdominal exploration. In an established case the exact age of the fœtus is difficult to determine, and the onset of spurious labour is not good evidence that full term has been reached. Spurious labour may commence at any time during the later months and continue for a varying time. The child never long survives its onset, the placental circulation being disturbed and hæmorrhage taking place into the gestation sac, rupture of the latter rarely occurring. It is extremely painful and exhausting to the mother, and the contraction of the abdominal muscles may perhaps contribute to the death of the child independently of interference with the placental circulation.

[1] Science and Art of Midwifery. [2] Lectures on Ectopic Gestation. [3] Ectopic Gestation. [4] Centralb. für Gunak, 1902. [5] Handbook of Obstetrics. [6] Brit. Med. Jour., July, 1903.

